# Altered Topological Properties of Gray Matter Structural Covariance Networks in Minimal Hepatic Encephalopathy

**DOI:** 10.3389/fnana.2018.00101

**Published:** 2018-11-28

**Authors:** Tian-Xiu Zou, Lilan She, Chuanyin Zhan, Yong-Qing Gao, Hua-Jun Chen

**Affiliations:** ^1^Department of Radiology, Fujian Medical University Union Hospital, Fuzhou, China; ^2^Department of Radiology, Fuqing City Hospital, Fuqing, China

**Keywords:** minimal hepatic encephalopathy, small-world network, graph theory, brain structural network, gray matter volume

## Abstract

**Background and Aims:** Liver cirrhosis commonly induces brain structural impairments that are associated with neurological complications (e.g., minimal hepatic encephalopathy (MHE)), but the topological characteristics of the brain structural network are still less well understood in cirrhotic patients with MHE. This study aimed to conduct the first investigation on the topological alterations of brain structural covariance networks in MHE.

**Methods:** This study included 22 healthy controls (HCs) and 22 cirrhotic patients with MHE. We calculated the gray matter volume of 90 brain regions using an automated anatomical labeling (AAL) template, followed by construction of gray matter structural covariance networks by thresholding interregional structural correlation matrices as well as graph theoretical analysis.

**Results:** MHE patients showed abnormal small-world properties of the brain structural covariance network, i.e., decreased clustering coefficient and characteristic path length and lower small-worldness parameters, which indicated a tendency toward more random architecture. In addition, MHE patients lost hubs in the prefrontal and parietal regions, although they had new hubs in the temporal and occipital regions. Compared to HC, MHE patients had decreased regional degree/betweenness involving several regions, primarily the prefrontal and parietal lobes, motor region, insula and thalamus. In addition, the MHE group also showed increased degree/betweenness in the occipital lobe and hippocampus.

**Conclusion:** These results suggest that MHE leads to altered coordination patterns of gray matter morphology and provide structural evidence supporting the idea that MHE is a neurological complication related to disrupted neural networks.

## Introduction

Previous studies have shown that brain structural abnormalities commonly occur in cirrhotic patients. Neuropathological studies have documented the loss of brain parenchyma due to cirrhosis (Kril and Butterworth, 1997). Among patients with hepatic dysfunction, neuronal cell loss (probably due to chronic portosystemic shunting and ammonia exposure) has been associated with liver failure (Butterworth, 2007). *In vivo* studies using computed tomography and magnetic resonance imaging (MRI) also reveal diffuse brain atrophy in cirrhosis (Tarter et al., 1986; Zeneroli et al., 1987, 1991; Iwasa et al., 2012), which progresses with advanced liver disease (Guevara et al., 2011).

Minimal hepatic encephalopathy (MHE) is a common complication of liver cirrhosis and is characterized by a wide range of mild neurocognitive impairments, such as slowing of psychomotor activity, attention deficits, impairment of memory, and decreased executive abilities (Bajaj et al., 2009), which in turn negatively influence daily activities and are often associated with poor prognosis (Stewart and Smith, 2007). Although MHE is traditionally considered to be a disease related to brain dysfunction, its neuropathological mechanisms remain unclear. New findings suggest that brain structural impairments may play another important role in MHE. Patients with MHE showed more serious brain atrophy as compared with those without MHE (Guevara et al., 2011). A previous study has suggested that structural alterations are associated with lower psychometric performance of cirrhotic patients (Amodio et al., 2003).

Despite the above findings in cirrhotic patients with MHE, previous studies have only focused on regional structural changes in cirrhosis and did not consider brain network-level architecture (i.e., topological organization). Recent progress in structural MRI analysis has facilitated the development of a human brain structural network model that is based on statistical correlations of morphological descriptors, including the thickness of the cortex or regional gray matter volume (RGMV; He et al., 2007; Wu et al., 2012). This type of brain network possesses small-world properties (He et al., 2007; Lv et al., 2010; that feature extensive local clustering and short path lengths that link each network node; Watts and Strogatz, 1998), thereby making it an attractive model for evaluating complex brain networks because it allows specialized, as well as integrated, information processing and maximizes information propagation efficiency using minimal wiring costs (Sporns et al., 2005; Achard and Bullmore, 2007). Gray matter structural associations have been shown to reflect neurological dysfunctions (Dazzan et al., 2004) and are correlated with functional connectivity (Bhojraj et al., 2010). Importantly, topological analysis of the brain structural network can provide new insights into various neurological diseases such as schizophrenia (Shi et al., 2012) and Alzheimer’s disease (Yao et al., 2010) and the normal aging process (Zhu et al., 2012), which all induce a disrupted integrity of brain functional networks. Moreover, the impaired small-world efficiency in the structural cortical network has been found to be associated with neurological disease progression (He et al., 2009). The abovementioned studies have also shown the study populations with either neurological diseases or aging exhibited considerable brain structural changes (Wen et al., 2011). Thus, it is hypothesized that the brain structural networks of MHE patients could exhibit large-scale topological alterations, as these patients have been found to have diffuse gray and white matter atrophy (Guevara et al., 2011; Iwasa et al., 2012; Montoliu et al., 2012).

Actually, MHE has been demonstrated as a neurological disease that is related to abnormal brain networks. MHE patients could exhibit altered functional connectivity involving various intrinsic brain networks, including those of dorsal attention, visual, auditory and default-mode (Qi et al., 2012). In addition, changes involving whole-brain functional connectivity have been reported in MHE patients (Zhang et al., 2012). Notably, based on the resting-state fMRI, it has been suggested that MHE is associated with decreased brain small-world network efficiency (Hsu et al., 2012). In these contexts, we aimed to make the first investigation on the topological alterations involving brain structural covariance networks in MHE patients, which can provide morphological insights into MHE mechanisms that may coincide with functional findings.

## Materials and Methods

### Subjects

The Research Ethics Committee of the Fujian Medical University Union Hospital, China approved this study, and each study participant provided written informed consent. This study involved total of 22 MHE cirrhotic patients and 22 healthy controls (HCs). The two groups were matched in terms of age, sex and education level. Table 1 shows the demographical data of the study participants. Liver cirrhosis was diagnosed based on biopsy (4/22) or individual history, biochemical and physical examination, as well as imaging such as ultrasound and computed tomography (18/22). Neuropsychological tests, namely, Psychometric Hepatic Encephalopathy Score (PHES) examination, were performed to assess MHE, which included a digit symbol test, number connection tests A and B, serial dotting, as well as line tracing. Details on how to diagnose MHE have been described previously (Chen et al., 2015). None of the participant developed neuropsychiatric disorders, received psychotropic medications, or was diagnosed with other uncontrolled endocrine or metabolic disorders (e.g., diabetes mellitus and thyroid dysfunction), or took excessive amounts of alcohol for 6 months before the study.

**Table 1:** Demographic and clinical features of the study participants.

	HCs subjects (*n* = 22)	MHE patients (*n* = 22)	*P* value
Age (years)	51.8 ± 6.5	51.3 ± 8.9	0.848
Sex (male/female)	15/7	17/5	0.498 (χ^2^ test)
Education level (years)	8.7 ± 2.2	8.8 ± 2.7	0.812
Etiology of cirrhosis (HBV/alcoholism/HBV+alcoholism/other)	-	13/4/2/3	-
Child-Pugh stage (A/B/C)	-	3/13/6	-
Final PHES score	0.7 ± 2.2	−8.2 ± 3.0	<0.001
Number connection test A (s)	37.0 ± 12.3	57.3 ± 17.3	<0.001
Number connection test B (s)	63.8 ± 29.2	134.2 ± 62.3	<0.001
Serial dotting test (s)	43.0 ± 7.6	65.3 ± 18.5	<0.001
Digit symbol test (raw score)	45.2 ± 9.3	26.9 ± 8.5	<0.001
Line tracing test (raw score)	115.8 ± 16.1	192.3 ± 47.3	<0.001

### MRI Acquisition

MRI scanning was performed using a 3.0 T scanner (Siemens, Verio, Germany). Three-dimensional T1-weighted sagittal images of magnetization-prepared rapid gradient echo (MPRAGE) were obtained using the following settings: TR = 1.9 ms, TE = 2.48 ms, FOV = 256 mm × 256 mm, matrix = 256 × 256, flip angle = 9°, slice thickness = 1.0 mm, 176 slices.

### Measurements of Regional Gray Matter Volume (RGMV)

The structural images were preprocessed using a voxel-based morphometry (VBM) toolbox as implemented in Statistical Parametric Mapping software (SPM8)^1^. Structural images were segmented to the GM, white matter, as well as cerebrospinal fluid. High-dimensional normalization was performed using the diffeomorphic anatomical registration using exponentiated lie algebra (DARTEL) approach (Ashburner, 2007) and then the segmented images were submitted to the Montreal Neurological Institute (MNI). We applied tissue deformation for modulation of the segmented GM images. Then, the entire gray matter was parcellated into 45 regions per hemisphere (a total of 90 regions, Table 2) as described by the automated anatomical labeling (AAL) atlas (Tzourio-Mazoyer et al., 2002), for RGMV estimation of each subject. Linear regression analysis was performed to exclude the effects of age, gender, as well as education level. The regression residuals were then substituted for the raw RGMV and designated as the corrected RGMV.

**Table 2:** Abbreviations of various automated anatomical labeling (AAL) regions.

Abbreviation	AAL region	Abbreviation	AAL region
AMYG	Amygdala	MOG	Middle occipital gyrus
ANG	Angular gyrus	SOG	Superior occipital gyrus
CAL	Calcarine fissure and surrounding cortex	OLF	Olfactory cortex
CAU	Caudate nucleus	PAL	Lenticular nucleus, pallidum
ACG	Anterior cingulate and paracingulate gyri	PCL	Paracentral lobule
DCG	Median cingulate and paracingulate gyri	PHG	Parahippocampal gyrus
PCG	Posterior cingulate gyrus	IPL	Inferior parietal, but supramarginal and angular gyri
CUN	Cuneus	SPG	Superior parietal gyrus
IFGoperc	Inferior frontal gyrus, opercular part	PoCG	Postcentral gyrus
ORBinf	Inferior frontal gyrus, orbital part	PreCG	Precental gyrus
IFGtriang	Inferior frontal gyrus, triangular part	PCUN	Precuneus
ORBsupmed	Superior frontal gyrus, medial orbital	PUT	Lenticular nucleus, putamen
MFG	Middle frontal gyrus	REC	Gyrus rectus
ORBmid	Middle frontal gyrus, orbital part	ROL	Rolandic operculum
SFGdor	Superior frontal gyrus, dorsolateral	SMA	Supplementary motor area
SFGmed	Superior frontal gyrus, medial	SMG	Supramarginal gyrus
ORBsup	Superior frontal gyrus, orbital part	ITG	Inferior temporal gyrus
FFG	Fusiform gyrus	MTG	Middle temporal gyrus
HES	Heschl gyrus	TPOmid	Temporal pole: middle temporal gyrus
HIP	Hippocampus	TPOsup	Temporal pole: superior temporal gyrus
INS	Insula	STG	Superior temporal gyrus
LING	Lingual gyrus	THA	Thalamus
IOG	Inferior occipital gyrus		

### Construction of Brain Structural Covariance Network

**Figure 1:**
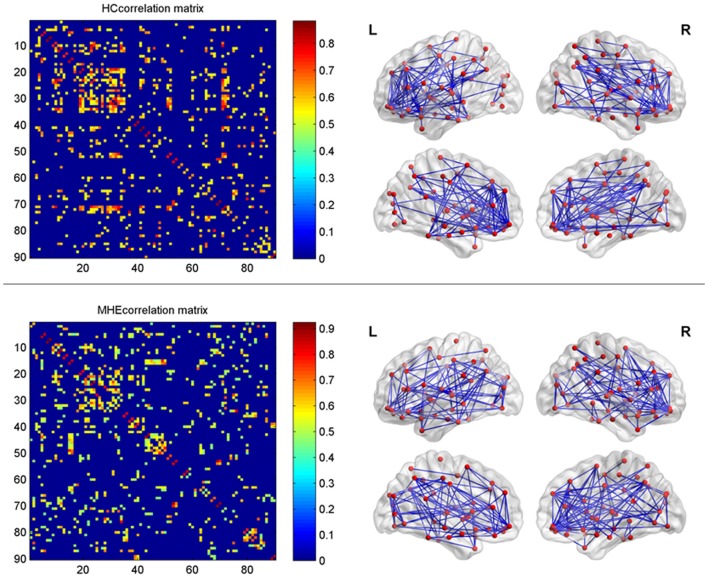
The interregional correlations matrix in healthy control (HC) and minimal hepatic encephalopathy (MHE) groups. The color bar represents correlation strength. These matrices indicate the maps thresholded at the minimum network density (=12%), wherein the networks depicted full connectivity (without fragmented nodes). The region number (from 1 to 90) represents automated anatomical labeling (AAL) areas, including AMYG.L(R), ANG.L(R), CAL.L(R), CAU.L(R), ACG.L(R), DCG.L(R), PCG.L(R), CUN.L(R), IFGoperc.L(R), ORBinf.L(R), IFGtriang.L(R), ORBsupmed.L(R), MFG.L(R), ORBmid.L(R), SFGdor.L(R), SFGmed.L(R), ORBsup.L(R), FFG.L(R), HES.L(R), HIP.L(R), INS.L(R), LING.L(R), IOG.L(R), MOG.L(R), SOG.L(R), OLF.L(R), PAL.L(R), PCL.L(R), PHG.L(R), IPL.L(R), SPG.L(R), PoCG.L(R), PreCG.L(R), PCUN.L(R), PUT.L(R), REC.L(R), ROL.L(R), SMA.L(R), SMG.L(R), ITG.L(R), MTG.L(R), TPOmid.L(R), TPOsup.L(R), STG.L(R), and THA.L(R). L and R indicate the left and right side, respectively. The right panel shows the graphical representation of the corresponding brain connectivity. The abbreviations for brain regions are presented in Table 2.

In the present study, the structural connections involving the whole-brain network were described using statistical correlations between pairs of the corrected RGMV. For every group, Pearson correlation coefficients between corrected RGMV were calculated across each study participant to generate the interregional correlation matrix (*N* × *N*, where *N* represents the number of gray matter areas, here *N* = 90). Then, the correlation matrix of every group was thresholded into a binary matrix and converted into an undirected graphical diagram (network; Figure 1). The constructed interregional correlation matrices were thresholded across various network densities (range: 0.12–0.50, with interval = 0.02; Singh et al., 2013). The minimum density was determined to guarantee that the brain structural network of the two groups was fully connected. For density >0.5, the network exhibited a higher degree of randomness (small-world index ~1.0; Singh et al., 2013). Finally, the graph theoretical methods were performed to analyze the resulting networks.

### Graph Theoretical Analysis

The Graph Analysis Toolbox (Hosseini et al., 2012), which integrates the Brain Connectivity Toolbox by Sporns and Rubinov for the quantification of network measures, was used in graph theoretical analysis.

To characterize the topological properties of brain structural networks, several key metrics were calculated, including the clustering coefficient (*C*_p_), characteristic path length (*L*_p_), and small-world parameters (i.e., normalized clustering coefficient (γ), normalized path length (λ), and small-worldness (σ); Watts and Strogatz, 1998; Rubinov and Sporns, 2010). *C*_p_ is the average clustering coefficients across all nodes of a network, wherein the clustering coefficient *C*_i_ of a node *i* pertains to the number of existing connections linking the neighbors of the node divided by all their possible connections. The *L*_p_ of a network pertains to the average distance of the shortest path involving all node pairs within the network, in which the shortest path represents the number of edges that connects two nodes. Previous studies have shown that brain functional and structural networks exhibit efficient small-world properties that allow the efficient transfer of parallel information using a relatively low cost (Watts and Strogatz, 1998; Achard and Bullmore, 2007; He et al., 2007). Thus, a brain network that features small-world properties possesses a higher *C*_p_ yet similar *L*_p_ compared to the null random networks (Watts and Strogatz, 1998), and a small-world network fulfills the following criteria: $\gamma ={\text{C}}_{\text{p}}^{\text{real}}/{\text{C}}_{\text{p}}^{\text{rand}}>1$, $\lambda ={\text{L}}_{\text{p}}^{\text{real}}/{\text{L}}_{\text{p}}^{\text{rand}}\sim 1$, and σ = γ/λ > 1, in which ${\text{C}}_{\text{p}}^{\text{rand}}$ and ${\text{L}}_{\text{p}}^{\text{rand}}$ represent the average clustering coefficient and characteristic path length of the matching random networks, respectively. In the present study, null random networks (number = 20) were generated from covariance matrices that match the distributional features of the observed covariance matrix based on the Hirschberger-Qi-Steuer algorithm, as described in previous studies (Zalesky et al., 2012; Singh et al., 2013).

For regional characteristics, the present study considered nodal degree as well as betweenness. The degree of a given node pertains to the sum of all connections situated between this node and the rest of the other nodes within the network. The betweenness of a specific node is described as the number of the shortest paths between a pair of nodes that run through this particular node. In the present study, the quantified nodal degree and/or betweenness were normalized using the average degree and/or betweenness of the network, respectively, followed by a comparison of two groups (Hosseini et al., 2012; Singh et al., 2013). The network hubs are the nodes that commonly interact with various other regions and allow functional integration as well as play a key role in instilling network resilience to insults. The nodes are thus regarded as the structural network hubs when these depict a higher degree (one standard deviation higher than the average network degree). The analysis of the regional network and hub was performed on the network that was thresholded at the minimum density for full connectivity.

### Statistical Analyses

The Graph Analysis Toolbox was used to conduct a comparison of network measurements between two groups. To test differences in topological parameters between groups, a permutation-based algorithm was employed (Bullmore et al., 1999; He et al., 2008). Here, a set of corrected RGMV data of one individual was randomly rearranged to that of another subject of any group, and then the correlation matrix was computed for the randomized group. Subsequently, a new binarized matrix was generated using a density threshold similar to that of real brain networks, followed by recalculation of the network parameters. We repeated this randomization procedure for 1,000 times using each threshold (density). Then, we computed for the network measures of all the networks at every density. We also calculated the differences in network measures among the newly randomized groups (using various network densities), thereby resulting in a difference permutation distribution under the null hypothesis. The entire set of 95 percentile points of every distribution was employed as the critical values in a one-tailed test of the null hypothesis using a type I error probability of 0.05.

In addition, the functional data analysis (FDA) was conducted to examine MHE-related differences in topological metrics. Here, we computed the sum of between-group differences in every network metric within a range of densities (0.12–0.50) that can be used as a summary scale for the topological assessment of brain structural networks, which does not require single-threshold selection. Subsequently, we performed a nonparametric permutation test using the FDA results to examine the significance of differences among groups (i.e., the observed summation).

## Results

**Figure 2:**
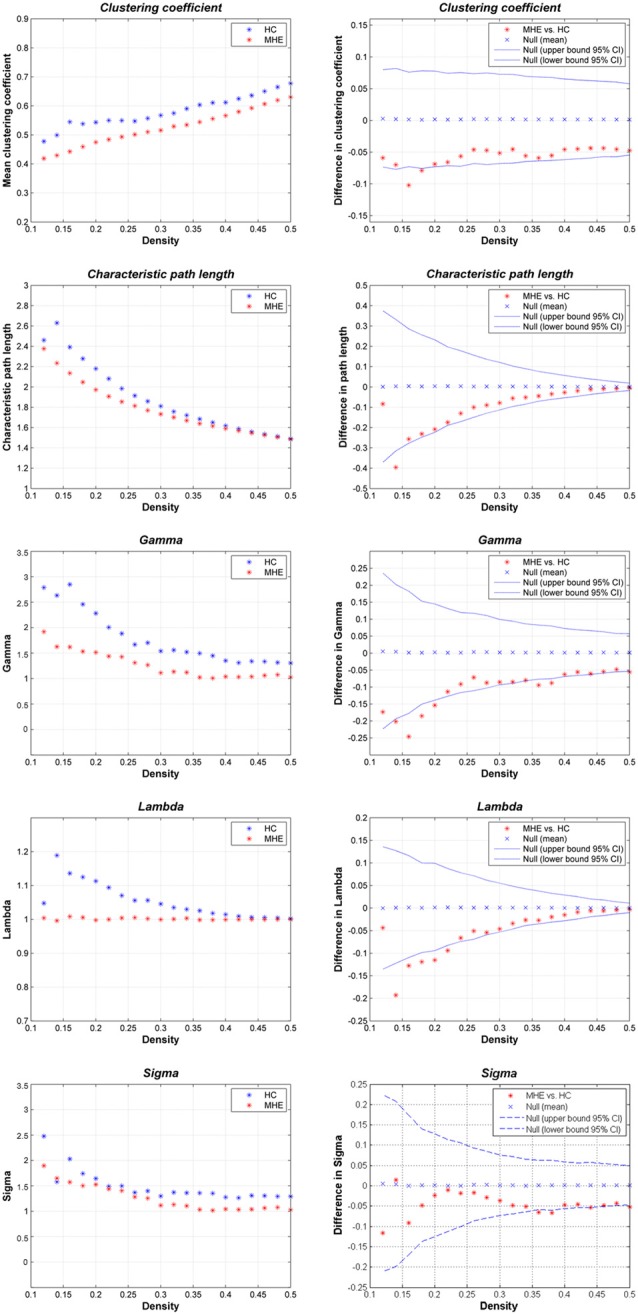
Small-world properties of the gray matter structural covariance network of MHE patients compared to HCs, using various network densities (0.12–0.50).

Figure 2 shows that the HC group demonstrated small-world network properties, i.e., considerable larger clustering coefficients and similar path lengths than those of the matched random networks. MHE patients showed lower *C*_p_, *L*_p_, γ, λ and σ. Statistically, FDA analysis indicated that MHE patients had significantly lower γ (*P* = 0.021), λ (*P* = 0.010) and σ (*P* = 0.048). For the non-normalized network measures, FDA analysis also indicated that the MHE group had decreased *C*_p_ (*P* = 0.030) and *L*_p_ (*P* = 0.043), as compared with HC group.

**Figure 3:**
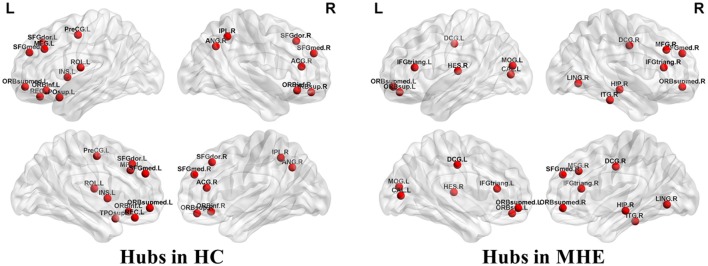
Network hubs in HCs and patients with MHE. The labeled nodes represent network hubs. The hubs that were common in two groups include the left ORBsupmed and right SFGmed. The hubs specific to HC include the bilateral SFGdor and ORBinf, left SFGmed, middle frontal gyrus (MFG), precental gyrus (PreCG), TPOsup, INS, ROL and REC and right ORBsup, ACG, IPL and ANG. The hubs specific to MHE patients include the bilateral IFGtriang and DCG, left MOG, ORBsup and CAL, and right ORBsupmed, MFG, HIP, LING, inferior temporal gyrus (ITG) and HES. The abbreviations for brain regions are presented in Table 2.

Figure 3 shows the hubs identified in each group. The number of hubs in the MHE group was less than that in the HC group. In the HC group, 17 regions were designated as network hubs, including eight association regions, eight limbic/paralimbic regions, and one primary region. These were primarily located in the prefrontal, parietal, insular and motor areas, which are involved in the executive control, default-mode, salience and motor networks (Barkhof et al., 2014). These hubs identified in our study agree with those of previous reports (Singh et al., 2013). The 15 hubs were identified in the MHE group, including seven association regions, six limbic/paralimbic regions, and two primary regions. These hubs were primarily in frontal/prefrontal, temporal, and occipital areas. All hubs in two groups are listed in Table 3. The hubs that were common in two groups include the left ORBsupmed and right SFGmed. The hubs specific to HC include the bilateral SFGdor and ORBinf, left SFGmed, middle frontal gyrus (MFG), precental gyrus (PreCG), TPOsup, INS, ROL and REC and right ORBsup, ACG, IPL and angular gyrus (ANG). The hubs specific to MHE patients include the bilateral IFGtriang and DCG, left MOG, ORBsup and CAL, and right ORBsupmed, MFG, HIP, LING, inferior temporal gyrus (ITG) and HES.

**Table 3:** List of network hubs.

Abbreviation	AAL region	Functional classification	Anatomical classification
**Hubs in HC group**			
ACG.R	Right anterior cingulate and paracingulate gyri	Paralimbic	Prefontal
ANG.R	Right angular gyrus	Association	Parietal
INS.L	Left insula	Paralimbic	Subcortical
IPL.R	Right inferior parietal, but supramarginal and angular gyri	Association	Parietal
MFG.L	Left middle frontal gyrus	Association	Prefontal
ORBinf.L	Left inferior frontal gyrus, orbital part	Paralimbic	Prefontal
ORBinf.R	Right inferior frontal gyrus, orbital part	Paralimbic	Prefontal
ORBsup.R	Right superior frontal gyrus, orbital part	Paralimbic	Prefontal
ORBsupmed.L	Left superior frontal gyrus, medial orbital	Paralimbic	Prefontal
PreCG.L	Left precental gyrus	Primary	Frontal
REC.L	Left gyrus rectus	Paralimbic	Prefontal
ROL.L	Left rolandic operculum	Association	Frontal
SFGdor.L	Left superior frontal gyrus, dorsolateral	Association	Prefontal
SFGdor.R	Right superior frontal gyrus, dorsolateral	Association	Prefontal
SFGmed.L	Left superior frontal gyrus, medial	Association	Prefontal
SFGmed.R	Right superior frontal gyrus, medial	Association	Prefontal
TPOsup.L	Left temporal pole: superior temporal gyrus	Paralimbic	Temporal
**Hubs in MHE group**			
CAL.L	Left calcarine fissure and surrounding cortex	Primary	Occipital
DCG.L	Left median cingulate and paracingulate gyri	Paralimbic	Frontal
DCG.R	Right median cingulate and paracingulate gyri	Paralimbic	Frontal
HES.R	Right heschl gyrus	Primary	Temporal
HIP.R	Right hippocampus	Limbic	Temporal
IFGtriang.L	Left inferior frontal gyrus, triangular part	Association	Prefontal
IFGtriang.R	Right inferior frontal gyrus, triangular part	Association	Prefontal
ITG.R	Right inferior temporal gyrus	Association	Temporal
LING.R	Right lingual gyrus	Association	Occipital
MFG.R	Right middle frontal gyrus	Association	Prefontal
MOG.L	Left middle occipital gyrus	Association	Occipital
ORBsup.L	Left superior frontal gyrus, orbital part	Paralimbic	Prefontal
ORBsupmed.L	Left superior frontal gyrus, medial orbital	Paralimbic	Prefontal
ORBsupmed.R	Right superior frontal gyrus, medial orbital	Paralimbic	Prefontal
SFGmed.R	Right superior frontal gyrus, medial	Association	Prefontal

**Figure 4:**
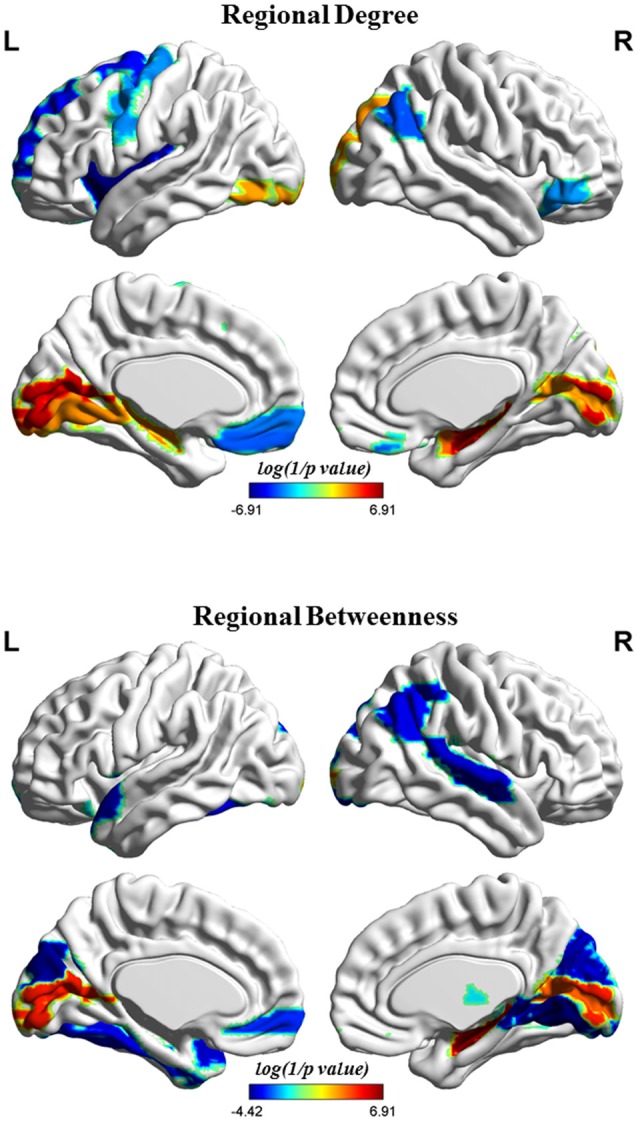
Brain regions depicting significant alterations in nodal degree and betweenness in the structural covariance network of the gray matter that was thresholded to a minimum density of full connectivity. The color bar indicates the log(1/*p* value). The cool color signifies regions with higher nodal betweenness or degree among HCs relative to MHE patients, whereas the warm color represents areas with greater nodal betweenness or degree in patients with MHE compared to the HCs. The abbreviations for brain regions are presented in Table 2.

Figure 4 shows the differences in regional network properties between two groups. Several brain regions with significant alterations in nodal degree and betweenness were detected in the structural covariance network of the gray matter that was thresholded to a minimum density of full connectivity. Compared to HC, the MHE group showed significantly lower nodal degree in the left SFGdor, ORBsupmed, PreCG, INS and REC and right ANG and ORBinf, whereas MHE patients had higher nodal degree in bilateral CAL and HIP, left LING and inferior occipital gyrus (IOG), and right superior occipital gyrus (SOG) and AMYG. In addition, the MHE patients showed significantly decreased nodal betweenness in bilateral CUN, left ORBsupmed, Fusiform gyrus (FFG) and TPOsup, and right ANG, IPL, LING, superior temporal gyrus (STG) and THA, and the MHE patients had significantly increased nodal betweenness in bilateral CAL and right HIP.

## Discussion

This study serves as the first investigation of the complex topological organization of brain structural networks in cirrhotic patients with MHE. Our results suggest that MHE patients exhibit an unoptimizable architecture involving the gray matter structural covariance network, as indicated by reduced clustering coefficients and characteristic path lengths and lower small-worldness parameters. These changes indicated a tendency to undergo additional random alterations in the brain structural network of patients with MHE, which shows less efficient communication of information across the whole brain. Similar to our results, a previous study has also revealed an anomalous gray matter structural network (with a decreased clustering coefficient and small-worldness) in the patients with cirrhosis (but only a few could be diagnosed as MHE; Lv et al., 2015). Thus, our findings provide morphological evidence that supports the concept that MHE is a neuropathological process associated with disruptive alterations to brain networks.

Previous analyses of functional connectivity networks have revealed that the neurocognitive deficiencies in MHE patients are associated with a deficiency in small-world properties, including more random and poor clustered architecture (Zhang et al., 2014; Jao et al., 2015). Studies have shown that the functionally linked networks reflect the underlying organization relating to the brain’s structural connectivity (van den Heuvel et al., 2009), and brain structural connectivity supports the functional network organization and various functional network graph features (Honey et al., 2009; Zimmermann et al., 2016). Therefore, it is expected that MHE patients have impaired topological organization in the brain structural covariance network, which may be considered as the neural substrate for the dysfunction of functional brain networks that subsequently contribute to neurocognitive impairment. The findings of this study indicate that the brain structural covariance network exhibits a more randomized configuration with lower *C*_p_, higher *L*_p_, as well as lower σ (Watts and Strogatz, 1998; Bullmore and Sporns, 2009) in patients with MHE, which agrees well with the results of earlier functional investigations (Zhang et al., 2014; Jao et al., 2015). The random networks involve fewer modularized information processing or tolerance of faults relative to small-world networks (Latora and Marchiori, 2001). Therefore, this explains why MHE leads to impaired small-world network efficiency functionality (Hsu et al., 2012), which indicates less efficient informational interactions between interconnected brain regions. Meanwhile, less fault-tolerance may imply an increase in vulnerability to cerebral metabolic disturbances (such as hyperammonemia-related edema and neurotoxic manganese deposition; Rovira et al., 2008) due to liver dysfunction. That may be the reason why once the disease gets to the stage of MHE, it is likely to progress to overt hepatic encephalopathy (HE), which has poor prognosis (Romero-Gómez et al., 2001; Stewart et al., 2007).

MHE patients had fewer hubs than those in the HC group (15 vs. 17) and showed a different distribution of network hubs from the HC group. They lost hub nodes mainly from the bilateral frontal lobe, right parietal lobe, as well as the left insular cortex, which are areas that are most frequently affected in MHE (Hsu et al., 2012; Zhang et al., 2012; Chen et al., 2013; Yang et al., 2018). Thus, our finding further indicated the selective vulnerability of these areas in the case of MHE. Although the mechanism about the loss of these hubs was not well understood, several pathological processes in MHE may contribute to the functional and structural alterations in these areas. For example, it could be implied that the alteration in cortical hub may be associated with the consequence of subcortical pathology (basal ganglia and cerebellum) due to hepatic dysfunction. The previous study has revealed abnormal cerebral blood flow/glucose metabolism in MHE, which is attributable to a redistribution of cerebral blood flow/glucose metabolism from the cortex to the caudate, thalamus, and cerebellum. Of note, the above hub-regions are considered as critical nodes that are responsible for high-level cognitive networks, such as executive activities, default-mode, as well as salience (Barkhof et al., 2014; Chen et al., 2016). Consistently, the reduction in nodal degree/betweenness in MHE was also found in the above networks. Therefore, the loss of these hubs may contribute to the common cognitive decline in MHE, such as executive dysfunction and attention deficit (Bajaj et al., 2009). However, the MHE group had new hubs, primarily located in the bilateral occipital lobe and right temporal lobe; consistently, they showed an increased degree/betweenness primarily in bilateral calcarine fissure and the surrounding cortex and bilateral hippocampus. As mentioned in previous studies (Hsu et al., 2012; Lv et al., 2015), these alterations implied the possibility that several hubs/nodes in the brain structural network undergo reorganization and compensate for the MHE-related declines in neurological function, such as visuo-spatial coordination and memory abilities (Ortiz et al., 2006; Bajaj et al., 2013; Ciećko-Michalska et al., 2013). This compensation mechanism would be helpful to delay the progression of HE disease.

This study has a number of limitations. First, we conducted a cross-sectional study. Future studies with longitudinal evaluation are recommended to directly test the progressive effects of HE on the topological properties of brain structural networks. Second, the topological measurements of the brain structural network can be identified by calculating the interregional correlations of RGMV among the participants in each group. The drawback of this approach in constructing a brain network is that it prevents the assessment of individual differences within network metrics, thereby making it difficult to explore the impact of unoptimizable network organization on MHE-related cognitive-behavioral outcomes. Third, the present study only assessed the topological features of the structural covariance network of the gray matter. The integrated analysis of functional as well as structural connectivity networks may generate novel insights into investigating the complex network properties of both healthy and diseased brains (Damoiseaux and Greicius, 2009; Zhang et al., 2011). Therefore, the further investigations involving multi-modal data, including T1-weighted, diffusion-based, as well as resting-state functional magnetic resonance images, should be performed to improve our knowledge of the topological features of complex brain networks in early stage HE.

In summary, our results revealed that MHE leads to the altered coordination patterns involving gray matter morphology. The loss of small-world topological features indicated a less efficient network organization even during the early stages of HE. The randomization alteration is an important characteristic occurring in the whole-brain network of MHE patients, which may contribute to the various neurological deficits, such as executive and attention dysfunction, and the motor and visual impairments. Our findings provide structural evidence that supports that MHE is a neurological complication related to disrupted neural networks.

## Author Contributions

H-JC, T-XZ and LS conceived and designed the study, acquired and analyzed the data and wrote the manuscript. H-JC, CZ and Y-QG contributed to data analysis. All authors have read and approved the manuscript.

## Conflict of Interest Statement

The authors declare that the research was conducted in the absence of any commercial or financial relationships that could be construed as a potential conflict of interest.

## Footnotes

^1^https://www.fil.ion.ucl.ac.uk/spm/

## Abbreviations

γ: normalized clustering coefficient
λ: normalized path length
σ: small-worldness
AAL: automated anatomical labeling
*C*_p_: clustering coefficient
FDA: functional data analysis
*L*_p_: characteristic path length
MHE: Minimal hepatic encephalopathy
PHES: Psychometric Hepatic Encephalopathy Score
RGMV: regional gray matter volume.
